# The Role of Personality Traits Toward Organizational Commitments and Service Quality Commitments

**DOI:** 10.3389/fpsyg.2020.00631

**Published:** 2020-05-15

**Authors:** Seungsin Lee, Jungkun Park, Ki-Joon Back, Hyowon Hyun, Suk Hyung Lee

**Affiliations:** ^1^Department of Global Business & Consumer, Konkuk University, Seoul, South Korea; ^2^Business School, Hanyang University, Seoul, South Korea; ^3^Conrad N. Hilton College of Hotel and Restaurant Management, University of Houston, Houston, TX, United States; ^4^College of Interdisciplinary Studies, SeoKyeong University, Seoul, South Korea

**Keywords:** hotel middle managers, personality traits, emotional labor, emotional exhaustion, organizational commitment, service quality commitment

## Abstract

Service providers personality traits is one of important determinants to deliver proper service to customers to make them satisfied in service delivery. Despite numerous studies on personality traits and emotional labor, little empirical work has been conducted to investigate the causal effects of hotel middle managers’ personality traits on their commitment to the hospitality industry. Thus, this study aims to examine the effects of hotel middle managers’ personality on two dimensions of commitments: organizational commitment and service quality commitment meditated by emotional variables: emotional labor and emotional exhaustion. The sample of this study consists of 266 department managers from full-service hotels in a metropolitan city in the Southern United States. The results confirmed the significant role of hotel middle managers’ personality traits, especially expressive personality, in organizational commitment and service quality commitment. Hotel operators should foster a work setting that consistently promotes congruent emotions via regular training and screening to reducing employees’ emotional exhaustion, increasing organizational commitment and service quality commitment, ultimately, reducing employees’ turnover intentions.

## Introduction

Researchers have agreed that understanding emotional factors and interrelations, such as emotional labor and emotional exhaustion, are essential for an employee’s well-being, ultimately creating employee commitment to the organization and increasing service quality. Numerous social and organizational behavior researchers have concluded that emotional labor may result in occupational stress and burnout, leading to job dissatisfaction and other job-related problems (Wharton and Erickson, 1995; Grandey, 2000; Kruml and Geddes, 2000; Cheung et al., 2011; Gursoy et al., 2011; Chen et al., 2012; Lee and Ok, 2014; Raman et al., 2016). Employee turnover rate has been increasing in the hospitality industry (Back et al., 2008; Jang and George, 2012). According to the data from the *Bureau of Labor Statistics’ Job Openings and Labor Turnover program* (2017), the restaurant and lodging industry turnover rate was 72.9% in 2016, which is 10% higher than that of 62.5% in 2013. One of the main reasons for high employee turnover rate is the emotional labor experienced by hospitality employees (Kim, 2008). Barnes (2001) further stated that the high turnover rate in the service industry is due to the recruitment of employees with unsuitable types of personality. One mechanism that has been recommended for reducing the high turnover rate or retaining valuable employees in the event of emotional labor is effective employee selection (Furnham et al., 2002; Kim et al., 2007; Riggio and Lee, 2007). More specifically, an effective selection procedure should look beyond technical skills and assess domains, such as personality traits and/or people skills (Shay and Tracey, 1997). There is consensus among researchers that people who have more expressive personalities are more likely to develop and utilize relationships that assist them in facing stressful situations (Jolson and Comer, 1997). Also, individuals whose personality traits positively associate with empathy, ego drive, patience, and enthusiasm, are more intent to seek enjoyment, self-expression, and perform organizational activities in order to experience the pleasure, stimulation, and joy inherent in the activity (Futrell et al., 1983; Yavas et al., 2010). In addition, Brown et al. (2002) stated that work orientation and personality traits were predictors for the quality of customer relations. Specifically, the quality of interpersonal relationship with supervisors, peers, and supervisees is critical, because it is both indirectly and directly related to employees’ job satisfaction (Furnham et al., 2002) and organizational commitment (Graf and Harland, 2005), as well as turnover intention (Donavan et al., 2004). O’Neill and Xiao (2010) found the significant direct and indirect effects of personality traits of hotel middle managers on emotional exhaustion. Their results were consistent with previous research that middle managers who had extroverted personalities tend to be largely buffered from emotional exhaustion.

Despite numerous studies on interplay of emotional labor, emotional exhaustion, and employee commitment, very little empirical work has been conducted to investigate the relationships among personality traits of middle managers as a critical component in the hospitality industry. Moreover, previous research (Cho et al., 2013) noted that some emotional variables have similar effects on employees’ commitment, but they have rarely been measured simultaneously in managerial levels. Most of previous studies dealing with emotional variables have focused on the perspective of service employees (e.g., Kim, 2008; Karatepe and Aleshinloye, 2009; Shani et al., 2014). Hotel managers are surrounded by constant stress due to the nature of business, which is a non-stop 24/7 operation. Although hotel middle managers may not be frequently required for direct contact with customers, their tough job environment that demands long hours of work, work overload, and swift handling of unpredictable situations involving both time and quality, entails a lot of job-related stress (O’Neill and Xiao, 2010). As an important decision-maker and problem solver, hotel middle managers are responsible for a burdensome task managing complaints from not only external customers who are hotel guests but also internal customers who are hotel employees (Ledgerwood et al., 1998). Specifically, Ledgerwood et al. (1998) claimed that perceived social and psychological workplace climate would lead to burnout more than actual works such as work shifts. Hence, emotional labor and emotional exhaustion that hotel managers experience could be different from frontline employees. Thus, the present study utilizes instrumentality and expressiveness as two-dimension of personality traits and aims to examine the effects of hotel managers’ two personality traits on organizational commitment and service quality commitment, mediating by emotional labor and emotional exhaustion. Most uniquely, the present study contributes to the interpretive discussion of how managers’ emotional states and two distinctive directional commitments are interrelated in the context of hotel service settings. The results of this study help ascertain its potential importance in organizations in terms of practical and academic insights into the area of employee selection and retention strategies.

## Literature Review

### Theoretical Background

#### Personality Traits

Personality traits have been regarded as significant determinants of individuals’ behavior, previous literature refers personality traits to cognitive (personal values), affective (attitudes), and behavioral patterns (behaviors) (Cattell and Tregaskis, 1965; Landers and Lounsbury, 2006; Huang et al., 2014). Some research has explored the significant causal effects of personality traits on consumer behavior (Bosnjak et al., 2007; Yoo and Gretzel, 2011), while several studies have utilized personality traits to identify their influence on employees’ behavior (Mount et al., 2006; Walumbwa and Schaubroeck, 2009). Several researchers have investigated five dimensions of personality traits in the industry that is often called the Big Five: Neuroticism, Extraversion, Conscientiousness, Agreeableness, Openness to experience (Goldberg, 1990; De Raad et al., 1998; Hahn et al., 1999; Somer and Goldberg, 1999). Raman et al. (2016) examined the impacts of the aforementioned five dimensions of personality traits on frontline employees’ emotional intelligence, emotional labor, emotional exhaustion, and counter-productive work behavior. They found out that three dimensions of personality traits (extraversion, agreeableness, and neuroticism) influence employees’ emotional intelligence and two dimensions of personal traits (conscientiousness and openness) influence counter-productive work behavior. In addition, there was an indirect relationship between emotional intelligence and emotional exhaustion through emotional labor. Meanwhile, Lamont and Lundstrom (1977) reported 16 different variables (i.e., dominance, endurance, social recognition, empathy, and ego strength) and found that only endurance, social recognition, and dominance have positive influence on managerial ratings of performance.

##### Instrumental and expressive personality traits

Bem (1974) suggested two dimensions of personality traits, instrumentality and expressiveness, which are closely related to specific dimensions of culture. Specifically, Spence (1993) stressed that instrumentality is associated with masculinity, while Yee and Lei (2012) found consistent relationships between femininity with expressiveness and masculinity with instrumentality. For instance, masculinity related (instrumental) traits are seen as characteristics of assertiveness, independence, and dominance. The femininity related (expressive) traits include compassion, warmth, tenderness, sympathy, and sensitivity. As consistent with the previous research, the relevance of instrumental and expressive personality traits to job performance has been revealed (Comer and Jolson, 1985; Jolson and Comer, 1992; McFarland and Kidwell, 2006; Luria and Kalish, 2013; Carretta et al., 2014). Jolson and Comer (1992) found that sales managers perceived a relationship between saleswomen’s instrumental and expressive traits and their effectiveness in performing six generally accepted functions of selling: prospecting, making contact and establishing rapport, probing for needs, stimulating desire, closing, and retaining both the sale and customer. Industry psychologists have recognized that personality traits make a difference in handling stressful work situations (Tokar et al., 1998; Maslach et al., 2001). Also, measuring personality traits indicate predictive measures for emotional outcomes in service settings (Tan et al., 2004).

Kim et al.’s (2007) results indicated emotional expression and management were dependent on individuals’ dispositional factors. Also, Maslach et al. (2001) argued that specific personality traits might make a difference in coping with job stress. A person with more expressive personality tends to handle stressful works rather smoothly than a person with instrumental personality due to warmth, sympathy, and tenderness nature of the personality (Tan et al., 2004). In addition, O’Neill and Xiao (2010) found that hotel middle managers who had registered high in expressive personality traits are more prone to emotional exhaustion. Although previous researchers have examined significant difference in the magnitude of relationship between two types of personality traits (instrumentality and expressiveness) and emotional exhaustion as well as emotional labor, it is hard to justify that a person is mutually exclusive on either personality traits. It is rather arguable that a person has dual personality traits so that the behavioral outcome can be dependent on which personality trait has more predictive power on emotional handling situations (Singelis, 1994).

#### Emotional Variables: Emotional Labor and Emotional Exhaustion

Emotional variables include emotional labor and emotional exhaustion. Emotional labor has been acknowledged as one of the core antecedents of emotional exhaustion (Glomb and Tews, 2004) and employees working in sectors with high emotional labor levels were not found to have higher levels of emotional exhaustion than those working low emotional labor level jobs (Wharton and Erickson, 1995; Schaubroeck and Jones, 2000). Emotional labor and emotional exhaustion comprise essential emotional components in determining not only the measurement of employees’ performance and job satisfaction in the workplace, but also predicting their commitment toward the organization and service quality. Affective events theory is relevant to explain the importance of emotions in the hotel industry (Walter and Bruch, 2009). The theory indicates the nature of the job and the requirements for emotional behavior and work attitudes (Weiss and Cropanzano, 1996). A combination of daily fluctuation of emotional stages that employees experience on the job, lead to positive and negative emotions that may further lead to work attitudes, such as job satisfaction, performance, and organizational commitment (Ashkanasy et al., 2002). Based on the literature, the following hypotheses are proposed:

H1: Hotel middle managers’ instrumental personality trait positively influences their level of emotional labor.
H2: Hotel middle managers’ instrumental personality trait positively influences their level of emotional exhaustion.
H3: Hotel middle managers’ expressive personality trait positively influences their level of emotional labor.
H4: Hotel middle managers’ expressive personality trait positively influences their level of emotional exhaustion.

#### Emotional Labor

Hochschild (2012) introduced the emotional labor concept as “the management of feeling to create a publicly observable facial and bodily display” (p. 7). Emotional labor has been widely described as a dynamic self-regulatory process that employees utilize to express their emotions by surface acting and deep acting, continuously adjusting and monitoring their feeling during interactions with customers (Gabriel and Diefendorff, 2015). The core principle of emotional labor is the artificial manipulation of emotions in order to satisfy the needs of the organization according to display rules (Qin et al., 2012). Due to the unique characteristics of the hospitality industry, inseparability of production and consumption, hospitality firms are emphasizing providing positive attitudes and emotions by prescribing both implicit and explicit display rules during service encounters (Lee and Ok, 2012). Hospitality employees are required to present politeness, sympathy, warmth, and friendliness during interpersonal interactions with their customers (Lee et al., 2016). Responding to the rules of the firm, employees may choose to behave in different ways: surface acting and deep acting. Surface acting refers to a display of emotions without the true associated feeling, whereas deep acting highlights efforts to change the inner feeling of an individual (Seery and Corrigall, 2009). For example, the employees’ behavior of smiling, by either surface or deep acting, is defined as an emotional labor strategy (Hennig-Thurau et al., 2015). Employees may choose to behave consistently with the display rules by hiding their true emotions. Some may try to alter their own emotional feelings and behave accordingly. Extant research claimed that surface acting causes negative outcomes such as job stress, job dissatisfaction, and burnout due to emotional dissonance (Lee and Ok, 2014) while deep acting causes positive outcomes such as increased job performance and job satisfaction (Lee and Ok, 2012). However, numerous previous studies had stressed the antecedent role of emotional labor on employee burnout, job dissatisfaction, and turnover intent, which is mainly created by the stress that employees face in a service industry (Jung and Yoon, 2014). Emotional labor becomes a major challenge when employees keep modifying or hiding their true emotions, which may result in employees’ emotional exhaustion, job dissatisfaction, poor performance, and turnover behavior (Griffeth et al., 2000).

Although most emotional labor studies in the hospitality industry focus on employees’ perceived emotional labor and its antecedents and consequences (Kim et al., 2007; Kim, 2008; Lee and Ok, 2012), it is more significant when it comes to middle managers. Just as hospitality employees experience emotional challenges during service encounters, middle managers may even have more challenges when they have to deal with both external and internal customers. Hotel managers must understand employees’ performance and make appropriate decisions in the service sector (Hanzaee and Mirvaisi, 2013). Based on the literature, the following hypotheses are proposed:

H5: The level of emotional labor for middle managers in the hotel industry positively influences their level of emotional exhaustion.

#### Emotional Exhaustion

Emotional exhaustion is one of the three dimensions of burnout (Maslach and Jackson, 1981). Emotional exhaustion has been identified as “a chronic state of emotional depletion as a consequence of prolonged exposure to work stressors (e.g., anxiety, fatigue, work-related depression)” (Baba et al., 2009, p. 25). It refers to depression, hopelessness, and feelings of entrapment (Kim et al., 2007; O’Neill and Xiao, 2010; Zhao et al., 2014). The preponderance of evidence suggests that emotional exhaustion is the earliest symptom of burnout (Ashill and Rod, 2011). Emotionally exhausted employees feel helpless, lose self-esteem, feel a lack of accomplishment, and develop negative attitudes toward customers, the organization, their job, and themselves (Cordes and Dougherty, 1993). The emotional exhaustion leads to job dissatisfaction, which could directly increase turnover intention (Zopiatis et al., 2014). Lapointe et al. (2011) stated that emotional exhaustion would positively relate to turnover and have significant negative influence on commitment. Cropanzano et al. (2003) found a linkage between emotional exhaustion and organizational commitment when examining hospital workers. Furthermore, previous research has found a significant and negative relationship between emotional exhaustion and organizational commitment (Babakus et al., 1999; Cropanzano et al., 2003; Ashill et al., 2009). Former research suggested that emotional exhaustion could be led by negative experiences of work and has damage impact on service quality (Bennett and Barkensjo, 2005). Emotional exhaustion has been acknowledged as a negative factor for job performance (Wright and Cropanzano, 1998). Kim and Ra (2009) explored the negative influence of job stress on service commitment. A research conducted by Jeon (2015) explored the negative relationships between emotional exhaustion and commitment to customer service among flight attendants. Although the relationship between emotional exhaustion and organizational commitment has been tested by many previous studies, the direct relationship between emotional exhaustion and service quality commitment still needs more research to test, especially for middle managers in the hotel industry.

#### Commitment: Organizational and Service Quality

The nature of employee commitment to their organization and commitment to service quality has been a topic of great interest in the human resources management literature. According to Good et al. (1988), organizational commitment develops slower than job satisfaction, which is one’s identification with the organization and loyalty to the organization. Classical views of organizational commitment include an attitudinal perspective and a behavioral perspective. Porter et al. (1974) also explored organizational commitment as the strength of an individual’s identification with, and involvement in, a particular organization.

On the other hand, Becker (1960) described organizational commitment as the tendency to engage in consistent lines of activity because of the perceived costs of doing otherwise. According to DeCotiis and Summers (1987), organizational commitment has a positive effect on performance. Imran et al. (2014) conducted a study, which indicates that the relationship between job satisfaction and performance is strongly positive: employee performance and attitudes toward work have positive relationship with organizational commitment. Boshoff and Mels (1995) reported strong positive relationship between organizational commitment and performance by measuring internal service quality. Tsai and Wu (2011) hold the opinion that organizational commitment functions as a mediator between internal marketing and service quality. The relative influence of employee commitment to the organization on delivering service has been considered in the context of service quality (Zeithaml et al., 1990).

**FIGURE 1:**
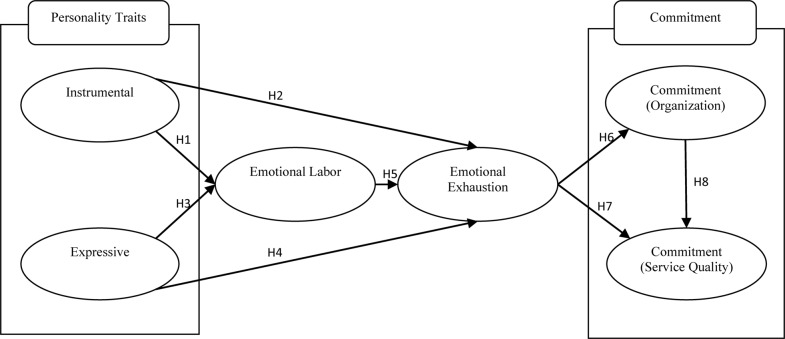
Conceptual model of personality traits and emotional labor.

Commitment to service quality also has been defined as an attitudinal commitment, which represents the employee’s dedication to providing quality service to customers (Ahmed and Parasuraman, 1994). Mowday et al. (1979) suggested that commitment to service quality is similar to organizational commitment. However, Hartline and Ferrell (1996) explored the increase in employees’ commitment to service quality aimed to improve the organization’s service quality rather than the employee’s commitment to the organization itself. Furthermore, previous research mentioned that the high level of service quality commitment of middle managers would have significant effects on employees’ commitment to service quality, enhancing customer satisfaction, and lower turnover rate (Mathieu and Zajac, 1990; Hartline and Ferrell, 1996; Schwepker and Hartline, 2005). In order to test the relations between organizational commitment and service quality commitment, the following hypothesis is proposed in this research (see Figure 1):

H6: The level of emotional exhaustion for middle managers in the hotel industry negatively influences their level of organizational commitment.
H7: The level of emotional exhaustion for middle managers in the hotel industry negatively influences their level of service quality commitment.
H8: Hotel middle managers’ organization commitment positively influences their service quality commitment.

## Methods

### Sample and Data Collection

In order to examine the hypothesized model, we conducted an online survey with approval of IRB. The sample of this study consists of middle managers from full-service hotels in metropolitan cities in the Southern United States. One thousand questionnaires were randomly distributed via Qualtrics online survey tool with screening questions that asking respondents’ position at the hotel at the beginning of the survey after consent form signed by respondents. Of those, 312 questionnaires were returned. A total of 266 usable questionnaires were obtained after list-wise deletion for a 26.6% response rate. Sample characteristics show 147 of the respondents were females. The average age of the respondents was 48.5 years old. The reported average monthly income was $6,266.60 (Table 1).

**TABLE 1:** Respondents’ socio-demographic characteristics.

**Variable**		**Frequency**	**Percent**	**Mean (*SD*)**
Age	<40 years of age	64	24.1	48.5 (12.0)
	40–49	68	25.6	
	50–59	84	31.6	
	>60 years of age	50	18.8	
	Total	266	100.0	
Gender	Male	115	44.6	
	Female	143	55.4	
	Total	258	100.0	
Income	<$1,000	52	23.4	6,266.6 (13,810.1)
	$1,001–$3,000	73	32.9	
	$3,001–$6,000	49	22.1	
	>$6,001	48	21.6	
	Total	222	100.0	

### Measurements

All items in this study were measured on seven-point Likert-type scales. For the purposes of this study, eleven personality traits items by Jolson and Comer (1997) were used to measure instrumental (e.g., I am assertive) and expressive (e.g., I am sensitive to the needs of others) personality traits with the reliability of 0.93. Emotional labor measurements were adopted from Kruml and Geddes (2000) study (e.g., To do my job well, I talk myself into showing emotions that are different from what I truly feel.) which provided the reliability of 0.89. Three items of emotional exhaustion scales (e.g., I feel burned out from my work) by Maslach and Jackson (1981) were modified with reliability of 0.93. Finally, two commitments, five items of organizational (e.g., I would accept almost any types of job assignment in order to keep working for this organization: Mowday et al., 1979), and three items of service quality commitment (e.g., I enjoy discussing quality-related issues with people in my organization: Schwepker and Hartline, 2005) were used with reliability of 0.93 for both. All measurements were pilot tested and pre-tested with a group of hotel managers (Table 2).

**TABLE 2:** Resources and measurements of variables.

**Construct**	**Measurement items**	**Resources**
Personality traits (instrumental)	1. I am independent. 2. I am assertive. 3. I have strong personality. 4. I am dominant. 5. I am willing to take a stand on issues. 6. I am aggressive	Jolson and Comer, 1997
Personality traits (expressive)	1. I am sensitive to the needs of others. 2. I am understanding. 3. I am compassionate. 4. I am warm. 5. I am gentle.	Jolson and Comer, 1997
Emotional labor	1. To do my job well, I talk myself into showing emotions that are different from what I truly feel. 2. When working with customers, I attempt to create certain emotions in myself that present the image my company desires.	Kruml and Geddes, 2000
Emotional exhaustion	1. I feel burned out from my work. 2. I feel fatigued when I get up in the morning and have to face another day on the job. 3. I feel emotionally drained from my work.	Maslach and Jackson, 1981
Commitment (organizational)	1. I talk up my organization to my friends as a great organization to work for. 2. I would accept almost any types of job assignment in order to keep working for this organization. 3. I feel this organization really inspires the very best in me in the way of job performance. 4. This is the best of all possible organizations for which to work. 5. I am extremely glad that I chose this organization to work for over others I was considering at the time I joined.	Mowday et al., 1979
Commitment (service quality)	1. I feel strongly about improving the quality of my organization’s services. 2. I enjoy discussing quality-related issues with people in my organization. 3. I really care about the quality of my organization’s services.	Schwepker and Hartline, 2005

### Reliability and Validity Test – Measurement Test

Table 3 shows that all of the alpha coefficients for the data exceed the minimum standard of reliability (Cronbach’s α = 0.70) recommended by Nunnally (1978) for basic research. This indicates the reliability of the measures. Following Anderson and Gerbing (1988) two-step approach, a measurement model was estimated before the structural model. Results of the confirmatory factor analysis on the key constructs were good: χ^2^_237_ = 328.073, *p* < 0.00; GFI = 0.909; NFI = 0.937; RMR = 0.081; CFI = 0.981; RFI = 0.926; IFI = 0.982; TLI = 0.978; RMSEA = 0.038), and all of the indicator loadings were significant (*p* < 0.01). As shown in Table 3, the average variances extracted were all above 0.50, indicating convergent validity. Discriminant validity exists when the proportion of variance extracted in each construct [AVE; ρ_*vc* (η)_] exceeds the square of the coefficient representing its correlation with other constructs (Fornell and Larcker, 1981). The results of confirmatory factor analysis along with mean, standard deviation, and the construct correlations between each construct are presented in Tables 3, 4.

**TABLE 3:** Reliability and validity tests.

Variable	Indicator	Loading	*t*-value	SMC	Cronbach-α	AVE	C.R
Personality traits (instrumental)	PI 1	1.27	14.60	0.69	0.931	0.70	0.93
	PI 2	1.32	15.57	0.77			
	PI 3	1.14	15.18	0.73			
	PI 4	1.39	16.67	0.86			
	PI 5	1.33	13.45	0.60			
	PI 6	1.00	–	0.58			
Personality traits (expressive)	PE 1	1.03	14.88	0.65	0.894	0.63	0.79
	PE 2	0.95	13.39	0.56			
	PE 3	1.00	14.37	0.62			
	PE 4	1.08	14.78	0.64			
	PE 5	1.00	–	0.68			
Emotional labor	EL 1	1.06	9.48	0.77	0.844	0.73	0.64
	EL 2	1.00	–	0.69			
Emotional exhaustion	EE 1	1.01	19.96	0.75	0.926	0.81	0.77
	EE 2	1.09	22.98	0.89			
	EE 3	1.00	–	0.79			
Commitment (organizational)	CO 1	0.90	18.83	0.68	0.930	0.73	0.85
	CO 2	1.00	–	0.83			
	CO 3	0.97	20.19	0.73			
	CO 4	0.99	20.90	0.75			
	CO 5	0.85	18.37	0.67			
Commitment (service quality)	CS 1	1.02	26.05	0.83	0.934	0.83	0.92
	CS 2	1.00	–	0.91			
	CS 3	0.88	22.83	0.75			

**Goodness-of-fit: $\chi_{237}^{2}$ = 328.073, p < 0.00; GFI = 0.909; NFI = 0.937; RMR = 0.081; CFI = 0.981; RFI = 0.926; IFI = 0.982; TLI = 0.978; RMSEA = 0.038.**

**TABLE 4:** Construct means, standard deviations, and correlations.

**Construct**	**PI**	**PE**	**EL**	**EE**	**CO**	**z**
PI	1.00					
PE	–0.26	1.00				
EL	0.04	0.24	1.00			
EE	–0.01	0.19	0.42	1.00		
CO	0.18	–0.01	–0.19	–0.52	1.00	
CS	0.29	–0.07	–0.04	–0.21	0.43	1.00
Means	5.81	3.76	3.57	3.36	5.34	6.21
S.D	0.90	1.25	1.65	1.82	1.37	1.06

*PI, Personality Traits (Instrumental); PE, Personality Traits (Expressive); EL, Emotional Labor; EE, Emotonal Exhaustion; CO, Commitment (Organizational); CQ, Commitment (Service Quality).*

### Structural Modeling Test

Table 5 summarizes the results of the structural equation model of this study. The fit indices of the research model shows goodness-of-fit: χ^2^_24__3_ = 364.297, *p* < 0.00; GFI = 0.901; NFI = 0.930; CFI = 0.975; RFI = 0.920; IFI = 0.975; TLI = 0.972; RMSEA = 0.043. With regard to RMSEA, the fit index was 0.043, which was below the recommended cut-off level of 0.08 (Hair et al., 2006). With these multiple fit indices indicating a reasonable fit for this model, the results indicated that the data fit the model fairly well. The results of path coefficients supported all the hypotheses except for H1, H2, H4, and H7.

**TABLE 5:** Structural models results.

**Structural path**	**Coefficient**	***t*-value**
H1: Personality traits (instrumental) → emotional labor	0.254	1.717
H2: Personality traits (instrumental) → emotional exhaustion	-0.022	-0.146
H3: Personality traits (expressive) → emotional labor	0.389	4.126***
h4: personality traits (expressive) → emotional exhaustion	0.142	1.446
H5: Emotional labor → emotional exhaustion	0.518	6.309***
H6: Emotional exhaustion → commitment (organizational)	-0.453	-9.102***
H7: Emotional exhaustion → commitment (service quality)	0.018	0.397
H8: Commitment (organizational) → commitment (service quality)	0.335	6.027***

Goodness-of-fit: $\chi_{243}^{2}$ = 364.297, p < 0.00; GFI = 0.901; NFI = 0.930; CFI = 0.975; RFI = 0.920; IFI = 0.975; TLI = 0.972; RMSEA = 0.043.***p < 0.001.

H1 and H2, which proposed a causal relationship between sub-dimension of instrumental personality trait to emotional labor and emotional exhaustion, were not supported. As another sub-dimension of expressive personal trait toward emotionalexhaustion was not supported (H4), only expressive personal trait toward emotional labor was supported (H3: β = 0.389, *t* = 4.126, *p* < 0.001). Emotional labor toward emotional exhaustion (H5) was supported (β = 0.518, *t* = 6.309, *p* < 0.001). H6, regarding the effect of emotional exhaustion on organizational commitment, was supported (β = -0.453, *t* = -9.102, *p* < 0.001), however, H7, regarding the relationship between emotional exhaustion and commitment to service quality, was not supported (β = 0.018, *t* = 0.397). Finally, H8, which hypothesized the relationship between organizational commitment and commitment to service quality, was supported (β = 0.335, *t* = 6.027, *p* < 0.001).

## Discussion and Conclusion

This study provides researchers with several important additions to the information on personality traits, emotionally related constructs, and commitment in two aspects: organizational and service quality. First, with support for 4 of the 8 linkages, the results indicate a better fitting model for hotel services industry and expand previous research to the managerial level. This study also provides a foundation for researchers to deepen understanding of the role of personality traits and emotional variables for middle managers in the hotel industry. This study finds that expressive personality trait is the main predictor of emotional labor rather than instrumental personality trait. This could be explained by the nature of expressive personality, which is an integral part of building relationship with customers such as warmth, empathy, kindness, patience and so on (Jolson and Comer, 1997). According to McFarland and Kidwell (2006), employees with expressive traits are more likely to concern about other people’s feelings and thus are able to meet other customers’ needs for the sake of their own intrinsic reward. As expressive personality traits tend to focus on developing relationships with customers, managers who encompass expressive traits are more likely to become involved with emotional labor.

Surprisingly, both instrumental personality traits and expressive personality traits were not significant predictors of emotional exhaustion. However, personality traits indirectly influenced emotional exhaustion, which was mediated by emotional labor. Given that emotional exhaustion was not significantly related to commitment to service quality within this model, this study suggests that emotional exhaustion could indirectly affect commitment to service quality through commitment to organization. This finding is in line with the study of Cropanzano et al. (2003) that emotional exhaustion directly affects organizational commitment and indirectly affects effective work behaviors including work attitudes, job performance, and turnover intentions, which are mediated by organizational commitment.

When looking at integrated emotional variables, emotional labor and emotional exhaustion, this study provides several important insights to consider as this area of study advances. First, this study confirms the findings of the previous studies (e.g., Cho et al., 2013), even in the different industries and employee’s rank settings. Meanwhile, it also discloses the following discrepancies, which can be industry and employees rank specific: personality traits are not entirely related to emotion, in general; an instrumental personality trait has no significant link to emotional variables; emotional exhaustion does not fully influence overall commitment. All in all, the results confirm the impact of hotel middle managers’ expressive personality traits on their commitment, which is mediated by emotional variables.

### Implications

Overall, this study provides owners and upper-level managers in the hotel industry with strong evidence that managers’ emotions are pivotal when trying to increase organizational commitment. Given that integrated emotional variables, emotional labor and emotional exhaustion, are the main predictors of organizational commitment, ultimately enhancing commitment to service quality, hotel industry upper-level administrators or owners need to set realistic job expectations with appropriate emotional screening and training. When managers fail to manage emotions, these employees have an increased probability of failing to commit to service quality and the organization, in turn impacting line personnel. Thus, owners and upper-level management should consider focusing on developing methods or training to identify and hire employees who are adept at managing their emotions.

The results confirmed the importance of individual employees’ expressive personality traits, which can indirectly enhance organizational commitment to improving the commitment to service quality that mediated by emotional labor and emotional exhaustion. A selection process that can identify employees with high expressive personality traits should result in the hiring of those who can cope effectively with relationships in situations involving emotional labor. By implementing some personality traits testing during the selection process, employers can assess candidate’s expressive personality traits in terms of his or her unique relational competencies, interaction involvement, expression of genuine concern for customers, and friendliness.

Further, hotel industry organizations should foster a work setting that consistently promotes congruent emotions via regular training and screening. Understanding emotion and reducing employees’ emotional exhaustion is the next key area to focus on in increasing organizational commitment and ultimately increasing employees’ service quality commitment and reducing employees’ turnover intentions. In addition, the early identification of employee emotional exhaustion is critical because it significantly influences organizational commitment and, possibly, turnover intention. Upper-level hotel managers can regularly track their departmental manager records for sick leaves, injury rates, and absenteeism so that problems can be tackled at an early stage. Besides, senior management can adopt an open work culture policy to encourage subordinates to talk freely; this also leads to a better understanding of the staff’s feelings. For instance, general managers or senior executives could host a lunch for managers regularly and encourage them to express their views to management. Managers who identify these concerns and address them will be able to reduce emotional exhaustion.

### Limitation and Future Research

As with all research, this study has several limitations. This study focused on middle managers in the hotel industry only. While this allows for more control of industry-specific issues, the ability to generalize the outcomes of the study outside of the hotel industry or different ranks of employees within the industry is limited. Research should examine both the impact of personality traits on full scale and emotional intelligence scale as a new meaningful antecedent for outcomes of emotional variables on commitment. Although it was not intended to assess the effects of different types of middle managers in the study model, the result did not show any significant differences among managers from various departments. However, it is worthwhile to assess the possible moderating effects of managers from different scaled hotels (e.g., limited service, upper-upper scaled, luxury scaled hotels) whether there are different roles of personality and emotional labor that have impacts on employees attitude and behaviors toward the organization.

Cross-cultural studies could also investigate the possible moderating effects of culture in the relationships among study variables. Furthermore, this study could apply to different sectors of the hospitality industry that look at similar forms of emotional labor, such as the restaurant and airline industries using cross-sectional studies. Second, due to the nature of this study as a self-reported data-based study, the possible threat of common method variance may be present (Campbell and Fiske, 1959). For future studies, the multi-method process may be recommended to enhance the validity of the study. Also, as one-shot study, it would be valuable to conduct the future study as a longitudinal study in order to continually assess middle managers’ attitude changes. Finally, actual retention rates, quitting behavior, and other demographic information, such as educational background and religious identification should be included in future studies.

## Data Availability Statement

The datasets generated for this study are available on request to the corresponding author.

## Ethics Statement

The studies involving human participants were reviewed and approved by University of Houston’s IRB committee. The patients/participants provided their written informed consent to participate in this study.

## Author Contributions

All authors listed have made a substantial, direct and intellectual contribution to the work, and approved it for publication.

## Conflict of Interest

The authors declare that the research was conducted in the absence of any commercial or financial relationships that could be construed as a potential conflict of interest.
